# The complete chloroplast genome of *Desmodium styracifolium*

**DOI:** 10.1080/23802359.2020.1778564

**Published:** 2022-03-21

**Authors:** Le Thi Yen, Joonho Park

**Affiliations:** Department of Fine Chemistry, Seoul National University of Science and Technology, Seoul, South Korea

**Keywords:** Chloroplast genome, *Desmodium styracifolium*, phaseoloid legumes, monophyletic group

## Abstract

The complete chloroplast genome (cpDNA) of *Desmodium styracifolium,* an important medicinal herb for urolithiasis treatment, was sequenced and assembled from the whole genome data. The cpDNA of *D. styracifolium* is 149,155 bp in length with GC content of 35.2%. The genome has a quadripartite structure that is composed of a large single-copy (LCS, 82,476 bp) and small single-copy (SSC, 18,439 bp) separated by a pair of inverted repeats (IRa and IRb, 24,120 bp each). There are 128 genes in the chloroplast genome, including 83 protein-coding genes, 8 rRNA genes and 37 tRNA genes.

Desmodieae is a monophyletic group in the paraphyletic Phaseoleae group in the family Fabaceae. Many genuses in this contain high levels of antioxidant and anti-inflammatory utilized as traditional medicine in Asia (Govindarajan et al. 2007; Lai et al. 2010; Li et al. 2014). Recent studies have investigated the generic-level relationship of Desmodieae with related tribes, but using individual genes is insufficient for understanding of its evolutionary relationships. Therefore, the complete chloroplast genome (cpDNA) of *Desmodium* provides a rich information for phylogenetic characters gaining deep insight into the evolutionary relationship among phaseoloid legumes.

*Desmodium styracifolium* is one of the herbal medicines in Asia. The effects of *D. styracifolium* were observed in the treatment of calcium oxalate renal stone, kidney injuries, and hepatitis (Giang Phan et al. 2010; Zhou et al. 2018). Flavonoids, triterpenoids and polysaccharides were reported about these effects (Hirayama et al. 1993; Hou et al. 2018).

Chloroplast is an organelle found in plant and algae. cpDNA is a quadripartite structure consisting of two inverted repeat regions (IRa and IRb) dividing the circular genome into a large single-copy (LSC) and small single-copy (SSC) (Wang et al. 2018). cpDNA contain protein-coding genes and also the genes of rRNA and tRNA (Jiao and Guo 2014; Jansen et al., 2005). The cpDNA is highly conserved in terms of the gene structure and contents among plants, though it has undergone the gene rearrangements (Jansen et al. 2005). Hence, plastome provides a source of information to contribute to not only understanding chloroplast evolution but also phylogenetic analysis and comparative genomics in plants (Wolf et al. 2010).

The *D. styracifolium* leaves were collected from Kampot, Cambodia (10°40′15″N 104°9′31″E) and stored at National Institute of Biological Resources, Korea, Incheon (NIBRGR0000112251). The total genomic DNA was extracted following the optimized CTAB method as previous described (Sahu et al. 2012). The goal of this study is reporting the characteristics of the complete chloroplast genome of *D. styracifolium* obtained from the Illumina sequencing system (Illumina Inc., San Diego, CA).

The cpDNA of *D. styracifolium* is 149,155 bp in length composed of two IR regions of 24,120 bp that divide a LSC region of 82,476 bp and a SSC region of 18,439 bp. The overall GC content of the cpDNA is 35.2% and IR regions, LSC, SSC possess 42.1%, 32.8% and 28.1%, respectively. There are 128 individual genes in the genome with 83 protein-coding genes, 8 rRNA genes and 37 tRNA genes, in which 17 genes are duplicated in the IRs (*rps12, rpl2, rpl23, trnI-CAU, ycf2, trnL-CAA, ndhB, rps7, trnV-GAC, rrn16, trnI-GAU, trnA-UGC, rrn23, rrn4.5, rrn5, trnR-ACG, trnN-GUU*). A total of 12 intron-containing genes is shown, 10 containing one intron (*trnK-UUU, rps16, atpF, rpoC1, trnL_UAA, trnV-UAC, ndhB, trnI-GAU, trnA-UGC, ndhA*) and two (*ycf3* and *clpP*) containing two introns. The complete chloroplast genome sequence was deposited in GenBank under the Accession no. MN913536.

A phylogenetic tree was constructed based on the chloroplast genome of *D. styracifolium* and 5 species from NCBI database aligned with CLUSTALW. The software RaxMLv8.1.20 was used to construct maximum-likelihood tree. *Desmodium styracifolium* is closely related to *D. heterocarpon* (Figure 1).

**Figure 1.**
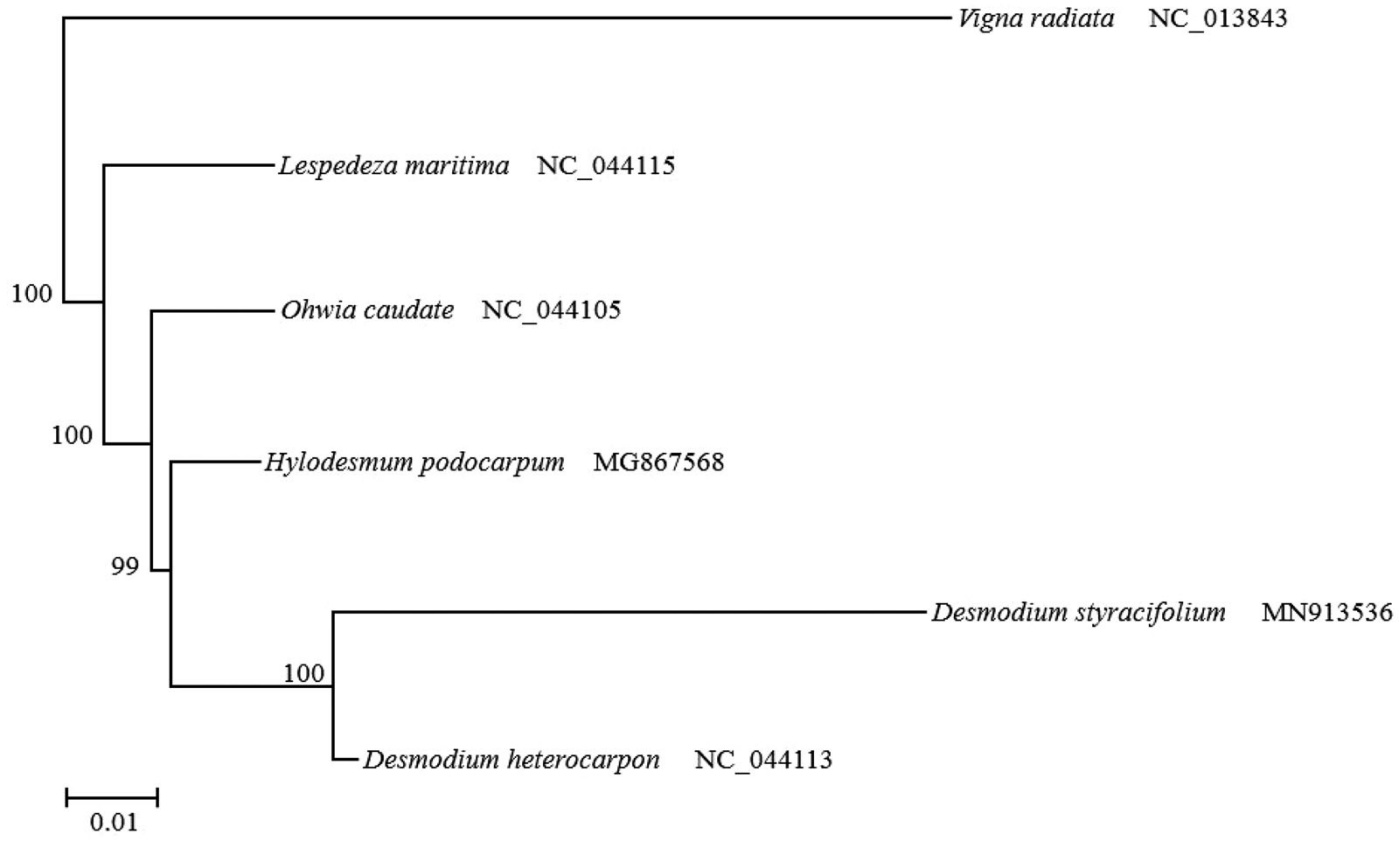
Phylogenetic tree constructed from complete genomes of 6 species using maximum-likelihood analysis with 1000 bootstrap replicates. Their accession number are as follows: *Desmodium heterocarpon* (NC_044113), *Hylodesmum podocarpum* (MG867568), *Lespedeza maritima* (NC_044115), *Ohwia caudate* (NC_044105), and *Vigna radiata* (NC_013843).

## Data Availability

The data that support the findings of this study are openly available in GenBank of NCBI at https://www.ncbi.nlm.nih.gov, reference number MN913536.
